# Phosphine-Stabilized Hidden Ground States in Gold Clusters Investigated via a Au_*n*_(PH_3_)_*m*_ Database

**DOI:** 10.1021/acsnano.2c07223

**Published:** 2022-12-30

**Authors:** Caitlin A. McCandler, Jakob C. Dahl, Kristin A. Persson

**Affiliations:** † Department of Materials Science, University of California, Berkeley, California94720, United States; ‡ Materials Science Division, Lawrence Berkeley National Laboratory, Berkeley, California94720, United States; § Department of Chemistry, University of California, Berkeley, California94720, United States; ∥ Molecular Foundry, Lawrence Berkeley National Laboratory, Berkeley, California94720, United States

**Keywords:** nanoclusters, gold, phosphine, ligands, DFT, high throughput, synthesis

## Abstract

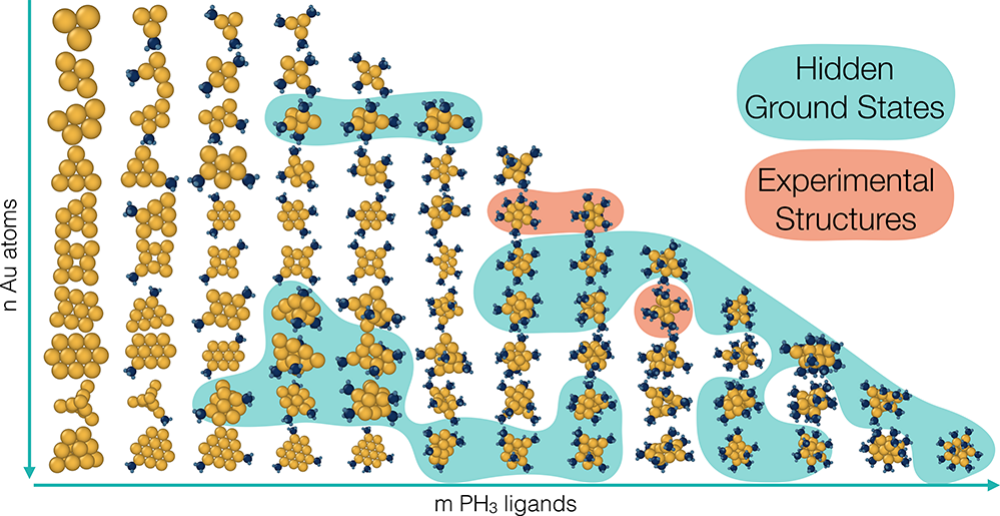

Nanoclusters are promising materials for catalysis and sensing due to their large surface areas and unique electronic structures which can be tailored through composition, geometry, and chemistry. However, relationships correlating synthesis parameters directly to outcomes are limited. While previous computational studies have mapped the potential energy surface of specific systems of bare nanoclusters by generating and calculating the energies of reasonable structures, it is known that environmental ions and ligands crucially impact the final shape and size. In this work, phosphine-stabilized gold is considered as a test system and DFT calculations are performed for clusters with and without ligands, producing a database containing >10000 structures for Au_*n*_(PH_3_)_*m*_ (*n* ≤ 12). We find that the ligation of phosphines affects the thermodynamic stability, bonding, and electronic structure of Au nanoclusters, specifically such that “hidden” ground state cluster geometries are stabilized that are dynamically unstable in the pure gold system. Further, the addition of phosphine introduces steric effects that induce a transition from planar to nonplanar structures at 4–5 Au atoms rather than up to 13–14 Au atoms, as previously predicted for bare clusters. This work highlights the importance of considering the ligand environment in the prediction of nanocluster morphology and functionality, which adds complexity as well as a rich opportunity for tunability.

## 1

Nanoclusters (NCs), a class of ultrasmall nanoparticles, are promising materials for catalysis, fluorescent sensors, bioimaging, nanomedicine, and precursors for nanoparticle synthesis. These materials measure less than 2 nm (<150 atoms) and exhibit molecular-like electronic structures as well as irregular atomic configurations. Their electronic and physicochemical properties are highly dependent on their composition, size, atomic configuration, and surface functionalization. Additionally, NCs exhibit large surface areas that are ideal for catalyzing reactions and discrete electronic states for optical applications.

It has been over 50 years since a gold cluster was first crystallographically resolved,^1^ and over the past two decades, nanoclusters have been synthesized with increasing diversity and identified with atomic-level precision.^2^ This has led to greater interest in *directing* the synthesis to design specific shapes and sizes. However, experimental nanocluster synthesis is time-intensive due to the difficulty of isolating nanoclusters in high enough purity for characterization and X-ray crystallography.^3^ Synthetic yields can be low due to poor selectivity, and transient, metastable intermediate clusters may be difficult to isolate or probe with *in situ* characterization.

Hence, improved predictions of stable structures, intermediate clusters, and possible reaction pathways provide a guide to possible synthesis products of a specific structure and size, as well as a fundamental understanding of how these nanoclusters form.

Solution-based growth is the preferred method to synthesize nanoclusters for several reasons. First, it offers the introduction of protective ligands to improve separation, storage, and size control.^4^ Second, solvated nanoclusters are required for solution-based processes, including photocatalysis, thin film processing, and drug delivery. Combinations of ligands can provide a variety of stable configurations and surfaces and result in highly variable catalytic performance.^5^ Importantly, ligation has been shown to be more effective at controlling the synthesis product than using kinetic control.^6^ It is clear that cluster thermodynamic stability and electronic structure and bonding are fundamentally influenced by the presence of ligands.

The structural and thermodynamic landscape of nanoclusters can be effectively explored using computational techniques. Indeed, global structure searching studies have extensively characterized the potential energy surface (PES) for bare gold clusters in the gas phase and have been highly successful in predicting the products of gold vaporization.^7−23^ Additional work has been done to predict the geometry of the ligand shell given the precise locations of the metal core atoms,^24^ to monitor the impact of SCH_3_ on Au_11_,^25^ PH_3_ on Au_13_^26^ and Au_8_,^27^ PH_3_ and Cl on Au_55_,^28^ and PH_2_(CH_2_)_*M*_PH_2_ (spacer *M* = 3, 5) on cationic Au_*n*_ (n = 7–11).^29^ However, to the best of our knowledge, there has been no work examining how ligation changes the sequence of stable cluster geometries, effectively stabilizing otherwise “hidden” ground states.

Here we present an extensive, grand canonical, data-driven study on ligated neutral gold clusters, spanning >10000 structures. We map the impact of ligation on the core gold structure (gold kernel) and analyze trends in preferred ligand binding sites, ground-state geometries, and hybridizations of gold–gold bonding. As a model ligand, we select phosphine, which exhibits weaker binding energies than thiolate ligands and is hence more suitable for catalysis^30^ and leads to less structural rearrangement. While clusters tend to adopt a positive charge according to the superatom model of stable electron counting,^31^ we consider only neutral clusters here. We show that, for the open system of phosphine and gold, phosphine ligation constitutes a crucial factor in the global search for stable nanocluster ground states.

## 2 Results/Discussion

### 2.1 Population Distribution

The ligation generation algorithm (see Computational Methods and Details) resulted in 10868 distinct Au_*n*_(PH_3_)_*m*_ (*n* ≤ 12) structures, which includes the initial set of pure gold structures, as well as an addition of the monomer, dimer, and experimental structures from the CSD.^32^ Due to the relaxation of a gold core in the presence of ligands, a highly diverse set of nanoclusters were produced. In total we obtained 4516 additional distinct geometries, as defined by having gold structures different from those of the original set. Details of the energy distribution and the number of structures calculated for each size are shown in Figure S2. Larger gold core sizes generated more structures due to the increased combinatorial space of ligand sites. It should also be noted that the initial gold kernels were the lowest energy species in the previously performed global energy search conducted to create the Quantum Cluster Database^21^ and as such are useful for the comparison with the PES of bare gold NCs.

The weaker binding energy and the single lone pair of electrons in phosphine ligands leads to a simple radial monodentate binding motif, which is easier to model than the complicated “staple” motif that thiolate ligands with three lone pairs available for binding adopt. Also, the weaker binding of phosphine causes less structural rearrangement of the gold NC core. Finally, a simple PH_3_ ligand can be substituted for the more complex but more commonly used triphenylphosphine (PPh_3_) to reduce the computational cost while still capturing some of the steric interactions, unlike the case for halides. However, we emphasize that some important differences to experimentally used ligands remain, including less steric hindrance, weaker binding energies, less electronegativity, and less polarizability.^33−37^ Further, bidentate phosphine ligands are not expected to be well represented with a simple PH_3_ ligand.

### 2.2 Grand Canonical Energy Formulation

The grand canonical energies of the structures were obtained according to
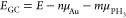
1where *n* represents the size of the gold cluster (number of gold atoms), and *m* represents the number of PH_3_ ligands in the cluster. All subsequent discussion of energy will refer to the grand canonical energy.

**Figure 1:**
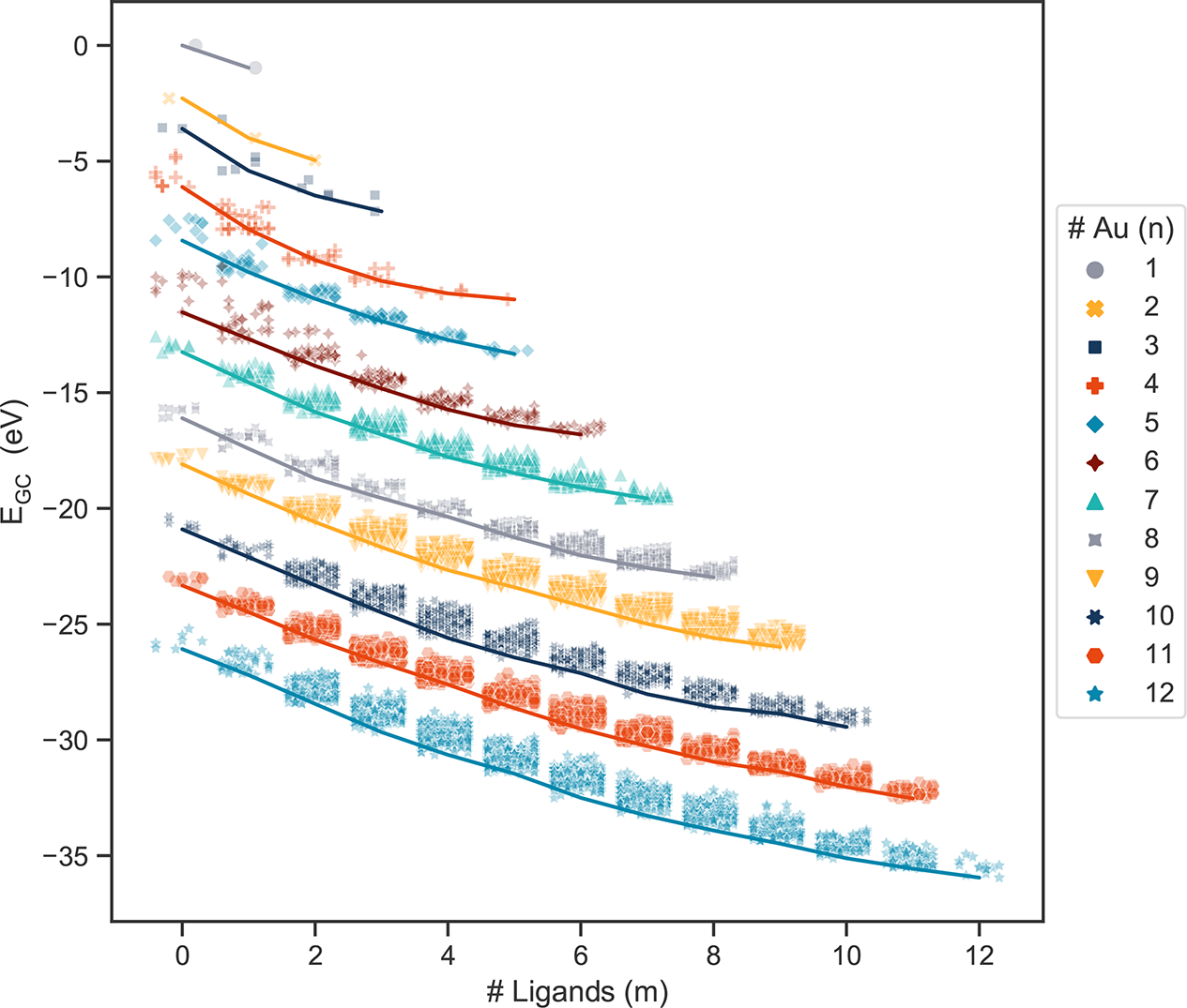
Grand canonical energies of the >10000 structures in the data set, including the experimental set. μ_Au_ = −0.29 eV and  = −15.62 eV. The slopes of the guide lines indicate the ligand binding energies. The ligand binding energies are given in Table S1 in the Supporting Information.

The chemical potentials of gold and phosphine, μ_Au_ and  were calculated with DFT as the total energy of a gold atom and a phosphine complex under vacuum and were obtained as −0.29 and −15.62 eV, respectively. The grand canonical energies of all structures in the data set are shown in Figure 1, as a function of the number of bonded ligands.

We note that there is likely a window of relevant chemical potentials, accessible by tuning the composition and concentration of the solution. For example, by applying the entropy correction of phosphine in the gas phase at 300 K provided in the NIST database,^38^ the resulting chemical potential of phosphine would decrease to −16.28 eV/PH_3_. Lower chemical potentials indicate a more stable environmental phosphine state, which competes strongly with the gold nanocluster ligation. We find that using the NIST gas-phase value results in sparingly ligated ground states, in contrast with experimental observations. Hence, we expect that most relevant solution chemical potentials of phosphine are higher than −16.28 eV. Using the higher DFT calculated , we find that ligand binding energy tapers off when sizes, *n*, are equal to the number of ligands, *m*. The ligand binding energy also decreases for larger structures of gold, meaning that a ligand is more stabilizing for smaller structures. A summary of the calculated ligand binding energies is included in Table S1. The most stable structures of all ground states with *n* = *m* were examined for ligand saturation; however, none of the structures, given the starting positions, were able to accommodate another ligand. Some experimentally realized structures, however, are able to bind more ligand head groups than gold atoms, typically by utilizing bidentate ligands (CSD IDs: 1541477,^39^ 1009716,^40^ 862706^41^).

### 2.3 Thermodynamic Stability Ranking

We find that the predicted ground states and thermodynamically ranked isomers depend strongly on the number of ligands bound to the nanocluster. This demonstrates that ligands stabilize some geometries more than others and that using the bare nanocluster energy rankings is not sufficient to predict the sequence of stable nanocluster structures as a function of size in an environment where ligating species are available.

**Figure 2:**
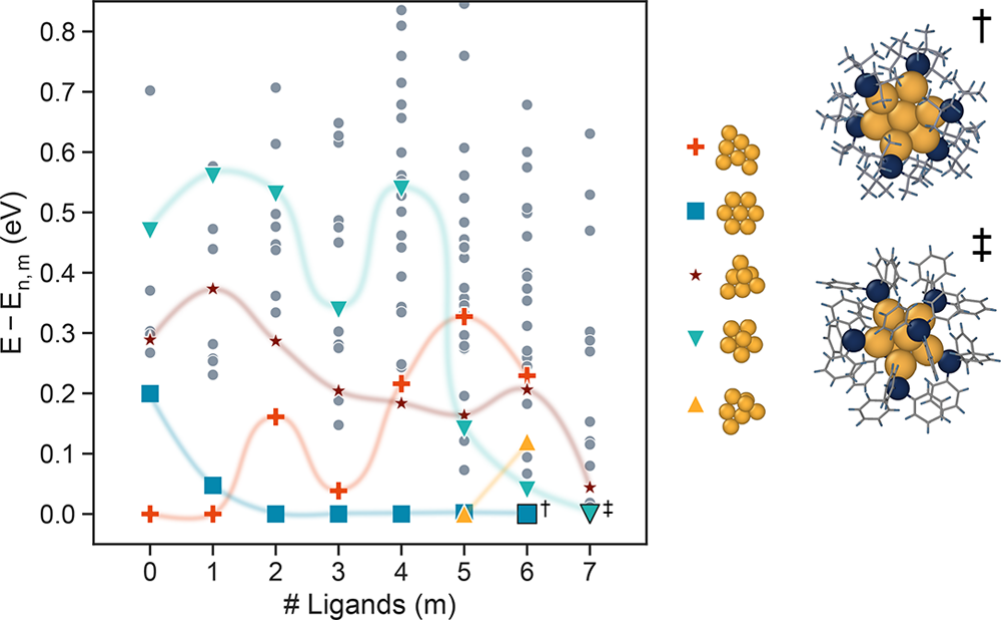
Energies of Au_7_(PH_3_)_*m*_ isomers for varying number of ligands, *m*, showing the strong influence of ligation. The energies are referenced to the minimum value for each *m*, *E*_7,*m*_. Each point represents a distinct gold kernel with the optimal ligand configuration for the given *m*; four gold kernels are highlighted in order to demonstrate how relative energies change with ligation. Experimental structures from literature sourced from the CSD are denoted with black outlines. Here, experimental structures are the lowest in the energy orderings for 6† (CSD ID: 2023935^42^) and 7‡ (CSD IDs: 668368,^43^ 1123094,^45^ 1123093,^44^ 1123095^46^) ligands.

In order to analyze the impact of ligands on the relative thermodynamic stability, structures with the lowest energy for a given gold kernel geometry were identified from each set of structures with *n* gold atoms and *m* ligands. Figure 2 shows the differences in the thermodynamic stability between the isomers and the ground-state structure with the same number of *m* ligands for a representative 7 gold atom kernel size. All other sizes between *n* = 3 and *n* = 12 are included in Figure S7. Relevant gold kernel geometries are identified and show large differences in calculated energies above the hull as a function of ligation. Importantly, we find that structures that have been observed experimentally (CSD IDs: 2023935,^42^ 668368,^43^ 1123093,^44^ 1123094,^45^ 1123095^46^) were correctly identified as ground states only with *m* = 6 and *m* = 7. Notably, these structures are 200 and 470 meV above the hull, respectively, in their bare, unligated states. Further, the ground-state bare Au_7_ cluster is not present in the Au_7_(PH_3_)_7_ set because the core gold geometry undergoes significant reorganization to accommodate 7 ligands; hence, there is no structure-matched equivalent ligated structure.

Below, we summarize some of the findings and comparisons to experimental synthesis products for each gold kernel size, *n*. In these comparisons, it is important to keep in mind that synthesis procedures involve crystallization and other postsynthesis techniques in order to improve selectivity. Also, any experimental structure that relaxed away from the geometry (and bonding) reported in the CSD was not considered as an experimental reference but is included in the data set.

#### 2.3.1 *n* = 4

The experimentally realized Au_4_ structure is a tetrahedron with 4 ligands (CSD IDs: 1206655,^47^ 1231463^48^). The bare tetrahedral structure is energetically unfavorable in computations, though it becomes notably more stable with ligation, improving agreement with experimental observations.

#### 2.3.2 *n* = 6

Two experimental clusters are considered (CSD IDs: 1120743,^49^ 1272194^50^). These structures are slightly distorted from a square-bipyramidal geometry and an octahedral geometry and were ranked fifth and ninth among the computed Au_6_(PH_3_)_*m*_, at 14 and 55 meV/Au above the most stable cluster, respectively. The two most theoretically stable gold kernels differ from the lowest energy experimental structure only by the rearrangement of 1 gold atom. Without ligation, the most stable Au_6_ structure is a planar triangle, but with ligation this planar structure is destabilized to 29 meV/Au above the most stable *n* = 6 structure.

#### 2.3.3 *n* = 8

One experimental structure retained its initial geometry during DFT relaxation and is considered here (CSD IDs: 1106337,^51^ 1106336^52^). It has 7 ligands (*m* = 7) with a hexagonal base structure and a relatively high energy, ranking 55th (72 meV/Au) within the Au_8_(PH_3_)_*m*_ structures. A lower chemical potential of phosphine, , would lower the relative energy of the Au_8_(PH_3_)_7_ experimental structure, as it is not fully saturated with ligands. The Au_8_ kernel that is the most stable in the bare system by 41 meV/Au can only accept 4 ligands before it breaks its 4-fold (square) symmetry or becomes highly energetically unfavorable.

#### 2.3.4 *n* = 9

Four different experimental Au_9_(PH_3_)_8_ structures were used as references, each having one central unligated Au atom. They are ranked 33rd (43 meV/Au) with an octahedral geometry (CSD ID: 1967410^53^), 51st (57 meV/Au) with a “butterfly” geometry (CSD IDs: 615444,^54^ 687192,^55^ 1273985^56^), 52nd (57 meV/Au) with a “crown” geometry (CSD IDs: 690419,^57^ 690422,^58^ 615445,^59^ 690418^60^), and 84th (81 meV/Au) with a “distorted crown” geometry (CSD IDs: 1895800,^61^ 1895797^62^) above the Au_9_(PH_3_)_*m*_ hull, respectively. The “distorted crown” experimental structure is likely high in energy with monodentate PH_3_ ligands, as it was synthesized with bidentate ligands.

### 2.4 Hidden Ground States

**Figure 3:**
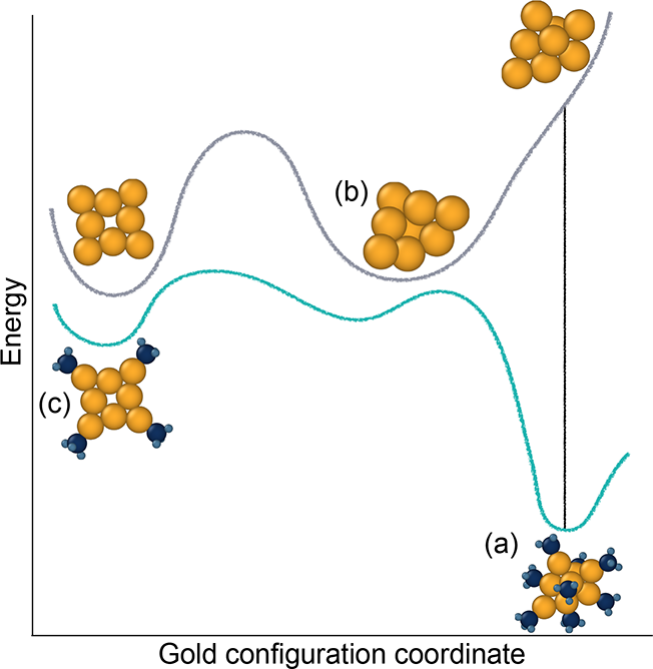
Illustration of the concept of hidden ground states, where the energies for bare gold and ligated gold are sketched as a function of a schematic gold configuration coordinate. Structure (a) is a hidden ground state, as it is the most stable Au_8_(PH_3_)_8_ structure in the data set but relaxes to structure (b) when its ligands are removed. Structure (c) is the most stable structure that can be achieved for the hollow square Au_8_ kernel that is the most stable in the bare PES.^27^

Many of the ligated structures exhibit gold kernels that are not local energy minima in the PES without ligation (i.e., there is no energy barrier between the geometry and a lower energy geometry). These structures are truly *hidden ground states*(63) such that they are dynamically unstable (e.g., a saddle point in the PES) in a pure gold system. An example of a hidden ground state identified here is the Au_8_(PH_3_)_8_ structure. In the case of this cluster, ligands stabilize a more 3D structure, labeled in Figure 3 as (a), which is *dynamically* unstable in its bare form and relaxes to a markedly different structure, labeled (b). Hence, the Au_8_(PH_3_)_8_ ground-state structure cannot be obtained by naive ligation of the bare ground state. Similarly, the ground-state Au_5_(PH_3_)_5_ manifests a 3D structure but spontaneously relaxes into a 2D geometry when the ligands are removed. The presence of such hidden ground states is an indication that a gold cluster PES is significantly affected by ligation. Hence, a pool of unligated metastable structures is likely to miss potential synthesis products and neglecting the effect of ligation leads not only to a shift in the relative energies of different geometries but also to overlooking specific ground states entirely.

To explore the prevalence of hidden ground states, we examined the ground state-structures—for all combinations of *n* atoms and *m* ligands—for metastability in their unligated state. From this analysis, 25 structures out of the 75 total ground states (3 ≤ *n* ≤ 12, 1 ≤ *m* ≤ *n*) and 6 out of the 10 fully ligated structures (*n* = *m*) were found to be hidden ground states. The full list of hidden ground state structures is included in Table S2.

### 2.5 Planar to Nonplanar Transition

**Figure 4:**
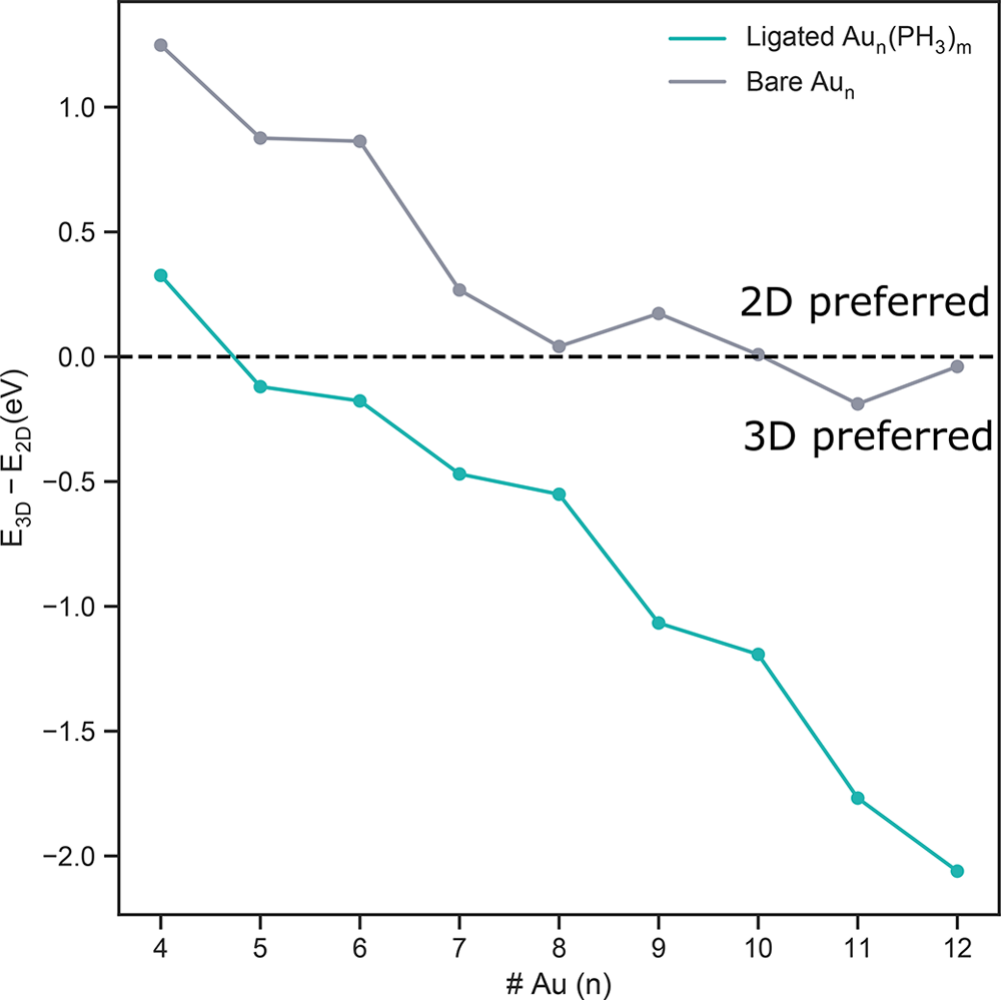
Energy difference(Δ*E* = *E*_3D_ – *E*_2D_) between the most stable 2D and 3D clusters as a function of cluster size, as predicted with and without ligation.

The size dependence of the planar to nonplanar transition of gold clusters is relevant for predicting structure–function correlations. However, most of the work on this topic has focused on bare clusters in the gas phase^8−22^ and predicts large planar to nonplanar transition sizes, up to 13–14 Au atoms.^20,21^ Here we find that the 2D to 3D transition in ligated systems occurs much earlier, with the transition occurring between 4 and 5 gold atoms. This finding better represents the early transition size of 3 to 4 gold atoms observed experimentally. Tetrahedral Au_4_ structures have been synthesized and characterized experimentally with bulky ligands: [Au_4_(P(mesityl)_3_)_4_]^2+^ and [Au_4_(P(*tert*-Bu)_3_)_4_]^2+^.^47,48^ We speculate that, if more bulky ligands were used in this study, such as PPh_3_, the size at which the transition occurs could be lowered further and recreate the experimental 2D to 3D transition size of 3 to 4 gold atoms. Figure 4 quantifies the degree to which the relative energy between 2D and 3D structures changes with the addition of ligands. Structures are defined to be planar (2D) if the average squared distance of gold atom positions to an optimal fitting plane is less than 0.1 Å. Positive values indicate that a 2D structure is preferred, and negative values indicate that a 3D structure is preferred.

We find 3D structures to be preferable for ligated structures for two main reasons: sterics and s–d hybridization. Both effects will be discussed in the following sections.

### 2.6 s–d Hybridization

Bare gold nanoclusters have been extensively investigated for the role of s–d hybridization in stabilizing planar configurations. While many argue that hybridization between the 5d and 6s orbitals is the key factor in stabilizing the planar gold structures,^64,65^ others have found that effects such as vdW interactions^18^ and d-electron delocalization^66^ are more important. Shafai et al. noted a shift in the d-band center in Au_13_(PH_3_)_12_ to lower energies for more 3D geometries, as well as increased Au s–d overlap with the P p-orbitals in 3D structures, while planar structures exhibit both bonding and antibonding contributions.^26^ Spivey et al. found that 3D geometries allow for better orbital mixing in Au_11_(SCH_3_)_*m*_ of S p-orbitals and Au d-orbitals.^25^

**Figure 5:**
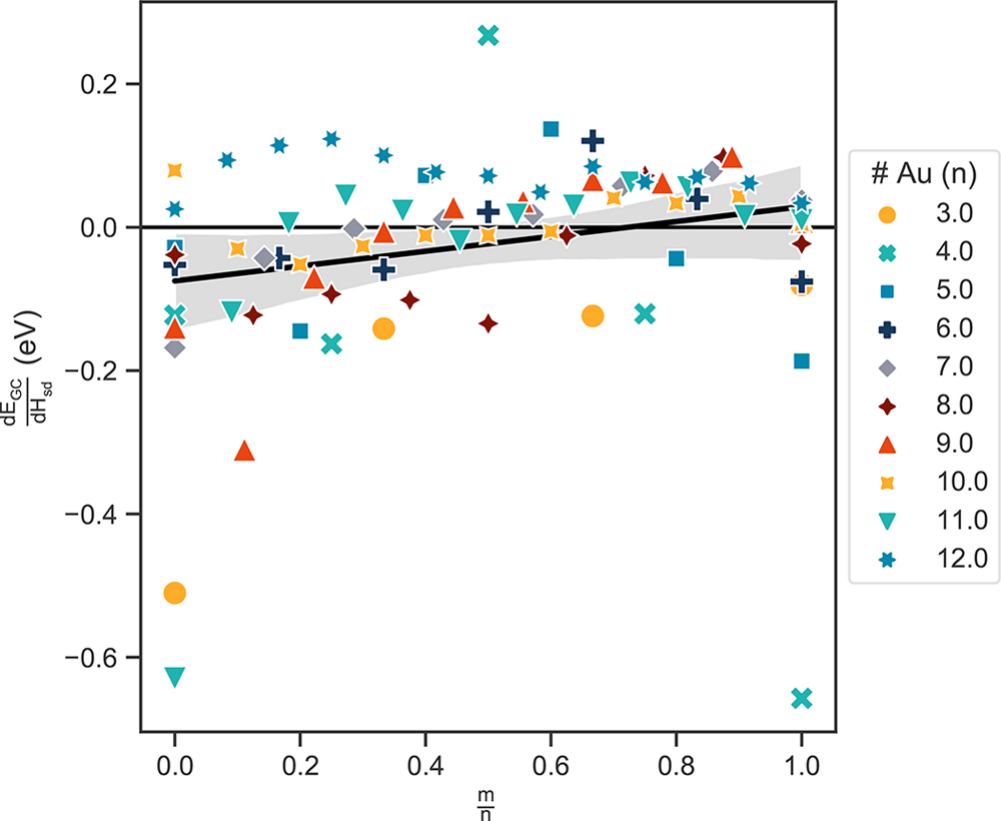
Derivative of grand canonical energies with respect to s–d hybridization index, , for each set of cluster sizes, *n* and *m*. The positive trend line indicates that clusters are destabilized with greater s–d hybridization at higher ligand coverage (ratio of ligands to gold atoms, ). Note that clusters with *n* atoms and *m* ligands with 4 or fewer data points were not considered due to poor statistics. s–d hybridization is responsible for the stabilization of 2D structures in the bare gold system. 3D structures are more stable when ligated, possibly having to do with this trend in reduced stabilization from s–d hybridization with more ligands.

Here we find a strong correlation between higher s–d hybridization and 2D configurations of gold, as suggested by the literature and exemplified in Figure S3 for a representative cluster size of *n* = 12. We further examine how the s–d hybridization is affected by ligation. Figure 5 shows the trend in stabilization from s–d hybridization, *H*_sd_ (as calculated per eq 3 in Section 4.2), as a function of ligation. As shown, there is a distinct increase in energy (destabilization) with respect to s–d hybridization, *H*_sd_, as a function of ligation. Given that phosphine exhibits a weaker binding energy as compared to other widely used ligands such as thiolates, these effects will likely be even more pronounced in systems with stronger binding energies.

### 2.7 Steric Effects

To analyze the local environment of the ligand binding sites and its impact on the cluster energy, we calculate the distances between neighboring ligands and evaluate the gold binding site topology. Analyzing the trends, we observe two similar but distinct steric effects. First, the steric repulsion of ligands in close proximity favors structures where the ligands maximize their distance from each other. Extrapolating to bulkier ligands, such as PPh_3_, it is likely that these effects will be amplified due to the larger radius of steric interaction. Second, we find that corner sites are preferred over edges and faces (see Figure S5). Comparing monoligated structures (i.e., Au_*n*_(PH_3_)_1_), we classify the structures by ligand binding site (corner or edge/face) by examining all bond angles between the binding gold atom and its neighboring gold atoms. Corner bonds are then defined as having Au–Au–Au (central Au is the binding Au) bond angles no greater than 140^*o*^. All structures with the same gold geometry were then compared according to their classifications. Indeed, corner-bound ligands were found to exhibit an average of 361 meV stronger binding energy than ligands bound to edges and faces of gold. Importantly, for larger sizes with saturated ligation, this preference for corners over edges leads initially planar structures to relax into 3D structures during geometry optimization in order to create more corner and edge sites.

### 2.8 Au_*n*_(PH_3_)_*m*_ Phase Diagram

To explore the phase space of most stable PH_3_-ligated Au clusters, we compute the grand canonical energy (eq 1) for a range of chemical potentials, μ_Au_ and , reflecting the ability to control these parameters through the concentration of precursors in solution. Additionally, the ligand binding energy, *E*_binding_, related to chemical potential according to eq 2, also correlates to the sterics and electron-donating properties of the ligand and can thus be changed by utilizing different chemical species.^67^
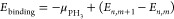
2

**Figure 6:**
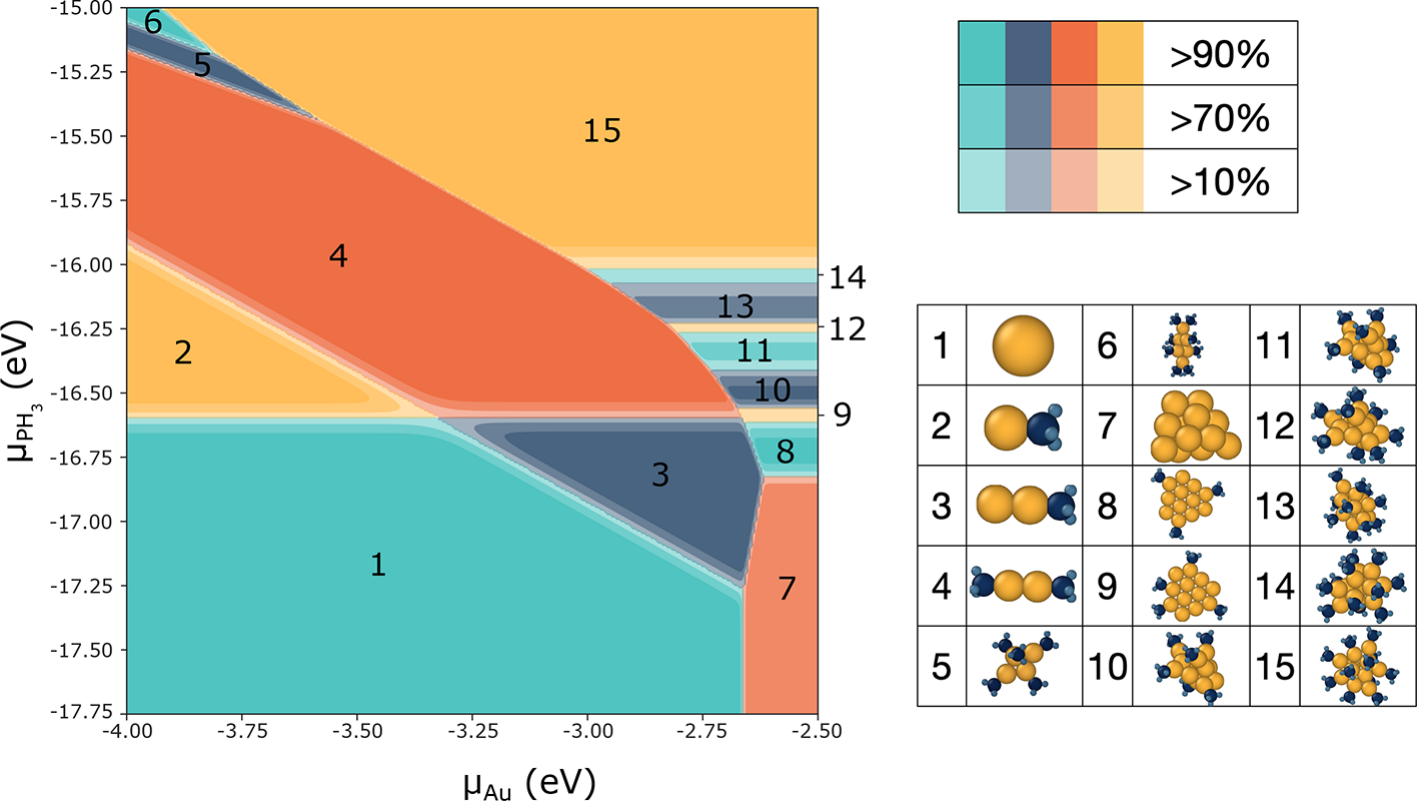
Au_*n*_(PH_3_)_*m*_ phase diagram created by finding the most stable structure in the data set for a range of chemical potentials. The stable species are shown on the right. The fraction of the solution product that they would expect to represent is calculated at 300 K via Boltzmann population statistics.

To estimate a synthesis yield based on relative energies, we assume that the structure population follows a Boltzmann distribution at 300 K. The results are shown in Figure 6, which prompts us to make the following observations. Changes between which structures are the most stable in the grand canonical ensemble only occur at very low chemical potentials of gold. The gold monomer and dimer occupy a large portion of the available phase space. However, at higher gold chemical potential, we find favorable conditions for small cluster formation, with a range of ligation as a function of phosphine chemical potential. At high phosphine chemical potential there is a strong stabilization of the largest, fully ligated cluster (here Au_12_(PH_3_)_12_), indicative of crystallization. We note that *n* = 12 is the limit of this data set, and it is likely that larger-sized clusters would successfully compete under these conditions.

A number of factors can influence the agreement between Figure 6 and experimental outcomes, and we emphasize that our findings should be taken as trends within chemical potential space, rather than pinpointing absolute values. For example, careful benchmarking work has shown DFT to exhibit errors in estimating the Au_2_ binding energy.^36^ An equivalent construction of a phase diagram included in Figure S6 shows the structures that might exist aside from the monomers and dimers.

### 2.9 Nanocluster Growth

**Figure 7:**
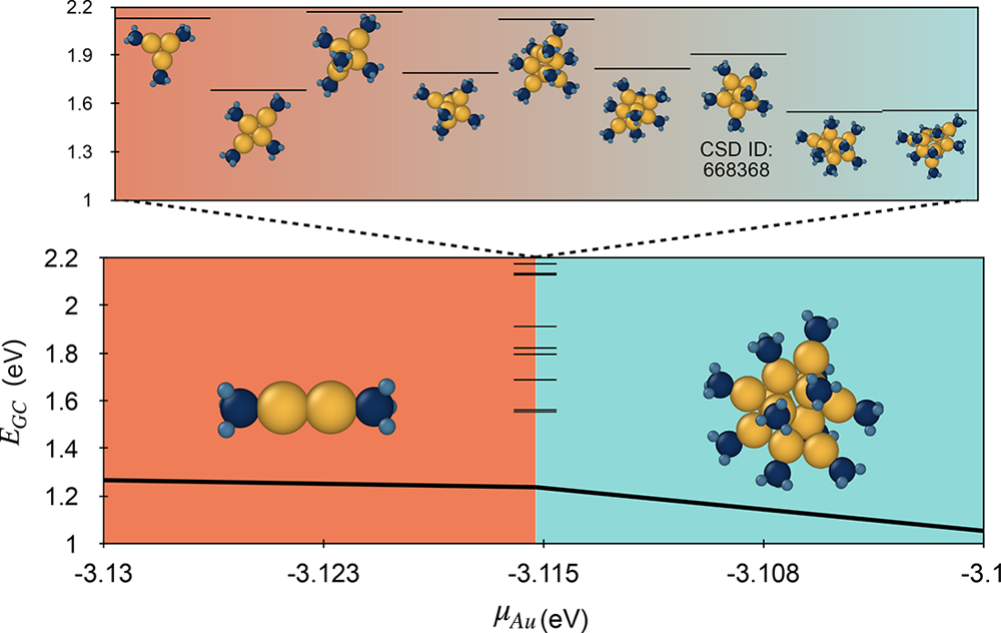
Possible transient intermediates in the transition between a small stable structure to a large stable structure. Assuming an increasing μ_Au_ and constant , set here to be −15.9 eV, the Au_12_ structure (right) would become more stable than the dimer (left), and some transient intermediate species (examples included in the inset) would be expected to exist in the growth process.

During a solution synthesis reaction of gold NCs, gold is reduced from Au(I) or Au (III) precursors. Hence, the concentration and thus the chemical potential of Au(0) are expected to monotonically increase, providing a measure of relative reaction progress. As a result, one would expect transient intermediate-sized structures to participate in the growth process from one cluster size to another. At transition points, i.e. chemical potentials at which the grand canonical energies of two stable structures Au_*n*1_ and Au_*n*2_ are equal, the set of clusters of intermediate size Au_*nx*_ (*n*1 < *nx* < *n*2) then present unstable intermediates, or transition-state structures. We can propose a model nanocluster growth mechanism to illustrate this concept using the lowest energy structures as example intermediates. We note, however, that many other structures are likely accessible as intermediates due to their relatively small energy differences and to temperature and kinetic effects. An example sequence of potential metastable intermediate clusters for a phosphine chemical potential of −15.9 eV is included in Figure 7. We note that one of the intermediates in this reaction pathway (size 7) has been successfully synthesized (CSD IDs: 668368,^43^ 1123094,^45^ 1123093,^44^ 1123095^46^).

While including ligation significantly improves the qualitative agreement between observed and predicted clusters, there are a few remaining questions and discrepancies. For example, calculated intermediates with odd numbers of gold atoms are systematically predicted to be higher in energy than the even-sized structures. Different odd–even behavior has been computationally reported, and we observe the same trend here (see Figure S3), with even-sized structures being predicted to be more stable than odd-sized structures;^9,11,68−70^ however, this behavior is not supported by experimental evidence. We do, however, note that even- and odd-sized clusters synthesized experimentally all exhibit even and odd cationic charges, respectively (Table S3). Thus, using a neutral (even) charge may preferentially favor even-sized clusters.

## 3 Conclusions

We generate and calculate—by a first-principles grand-canonical formalism—thousands of Au_*n*_(PH_3_)_*m*_ nanocluster structures to compare and analyze stability-promoting chemistry–structural trends. We find that the addition of phosphine ligands dramatically changes the bonding and hybridization in gold NCs such that the planar to nonplanar transition occurs between *n* = 4 and *n* = 5, earlier than predicted for the bare gold system and in improved agreement with experimental observations. The stabilization of 3D cluster geometries in the presence of ligation is rationalized by a combination of steric effects and s–d hybridization analysis. Furthermore, ligation stabilizes cluster geometries that are dynamically unstable in a pure gold system, resulting in a significant population of “hidden ground states”. These ground states manifest themselves in phase maps of cluster stability as a function of chemical potential, which lends insight into possible formation mechanisms. Our approach showcases the necessity of including ligands in calculations of nanocluster energies, as well as the predictive power of utilizing high-throughput DFT methods to map out potential gold nanocluster products and their formation pathways.

Simplifications employed here that are likely to further influence the stability of Au NCs include the use of PH_3_ instead of bulkier PR_3_ groups, the absence of solvation effects, and the neutral charge states. We expect that the treatment of charge will reduce the odd/even energetic disparity and that the increased steric repulsion of bulkier ligands will promote more compact clusters. Calculating the effect of steric bulk, charge, and solvation increases the computational demand beyond current capabilities for high-throughput electronic structure computation, and hybrid machine-learning models may be required for efficiently exploring this high-dimensional combinatorial chemical space. Future inclusion of these effects as well as increased cluster size is anticipated to guide practitioners to different experimental conditions and suggest formation mechanisms that can be empirically tested.

## 4 Methods/Experiments

### 4.1 Ligation Algorithm

**Figure 8:**
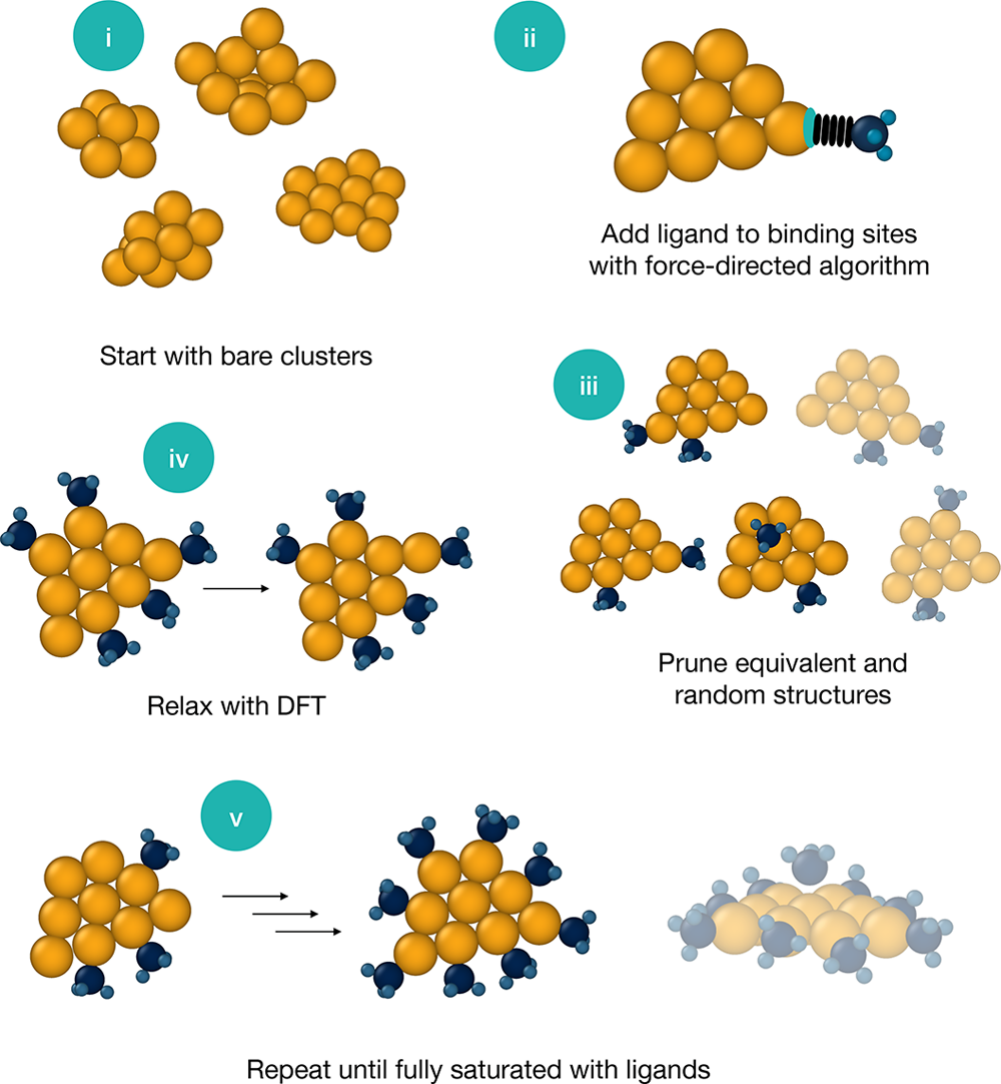
Ligation algorithm. Additional ligated clusters were generated by adding PH_3_ successively onto bare gold clusters.

A database of phosphine-ligated nanoclusters is generated from an initial set of bare structures as outlined below and illustrated in Figure 8. The algorithm is divided into steps i–v as follows (the same numbers are used in Figure 8).

(i) Initial structures. A group of previously predicted low-energy bare gold clusters is defined from the Quantum Cluster Database.^21^ Eighty-one structures between 3 and 12 atoms with low energies are taken as the initial set of gold cluster geometries.
(ii) Addition of ligands. A structure with an additional PH_3_ ligand is created for each possible ligand binding site. Binding sites are identified as gold atoms on the surfaces of the clusters that do not already have a bond to a PH_3_ ligand. Reasonable guesses for optimal ligand placements are made with a Fruchterman–Reingold force-directed algorithm, implemented by the *networkx* code, which treats the created bond between the Au and P as a spring and then adds electrostatic repulsion so that the new ligand is positioned away from the cluster.
(iii) Pruning. Each structure is compared to all others in the set, and any duplicate or symmetrically equivalent structures are removed. Structures are defined as duplicates if they have isomorphic bonding (Au–Au and Au–P bond cutoffs of 3.2 and 2.5 Å, respectively). In order to manage the combinatorial explosion of possible partially ligated structures, a random fraction of structures with duplicate gold kernels were removed under the assumption that those remaining constituted a sufficient sampling of possible partially ligated structures. To show an example as to why the structures required further pruning, we calculate the possible structures generated through the Au_12_(PH_3_)_*m*_ ligation. Each gold kernel could be ligated in 12! different ways. By subtracting all symmetry-equivalent ligation, this number could be reduced to about 4000 per single gold kernel. Given that the relaxation of gold kernels to different geometries generated approximately 400 distinct Au_12_ kernels, a full combinatorial evaluation would still require computation of about 1.5 million structures. Randomly discarding a fraction of the structures at each step reduced the number of structures calculated to a more manageable 3213 Au_12_ structures. The fraction of pruned structures correlated with the structure size and number of combinations of ligand configurations; as many as 90% of the largest structures (*n* = 12) with half-ligation (*m* = 6) were pruned, while none of the smaller (*n* < 8) or fully ligated (*n* = *m*) structures were pruned.
(iv) Relaxation. The structures are geometrically relaxed with DFT.
(v) Repeat and terminate. Steps ii–iv are repeated until the structures are fully saturated with ligands. Oversaturation is achieved when the last ligand does not bind to the cluster (Au–P distance >2.5 Å), and such structures are excluded in the final set. At this point, the algorithm terminates. We note that the outlined sequential procedure—which adds one ligand at a time and relaxes that cluster—does not target highly symmetrical ligated structures.

### 4.2 Computational Methods and Details

The structures of Au_*n*_(PH_3_)_*m*_ were geometrically relaxed with density functional theory (DFT). Additionally, 50 phosphine-stabilized gold structures from the Cambridge Structural Database (CSD) were computed with PH_3_ in place of their organophosphine (PR_3_) ligands. Twenty-one of those (10 different structures) maintained the same structures and gold bonding (Au–Au bond cutoff of 3.2 Å) during DFT geometry optimization and were taken as a set of reference experimental structures.

Spin-polarized calculations were performed with a plane wave basis set, as implemented in the Vienna Ab initio Simulation Package (VASP).^71^ A cutoff energy of 520 eV was applied for the plane wave basis set, and the electron–ion interactions were described by the projector augmented wave (PAW) method.^72^ The exchange and correlation energies were calculated using the Perdew–Burke–Ernzerhof (PBE) form of the generalized gradient approximation (GGA).^73^ The structures were provided at least 10 Å of vacuum along each direction to reduce self-interaction between periodic images.^74,75^ One *k* point, i.e., the γ point, was used in the cluster calculations, and Gaussian smearing was applied with a width of 0.2 eV. Spin–orbit coupling was not considered, given its computational cost and contradicting conclusions regarding its effect on the relative stability of bare Au clusters.^7,11^ A postrelaxation dispersion energy correction with zero damping (D3) was then applied.^76^

The s–d hybridization, *H*_sd_, is calculated according to the method described in the literature and reproduced in eq 3,^18,77^ where the variable *I* represents the atom index, *S* represents the spin state, *W*_*E*_ represents the occupation of the eigenvalue *E*, and *m* represents the index of d-orbitals. *w*_s_ and *w*_d_ are the weights of the projected wave function on the spherical harmonics within the Wigner–Seitz atomic radius around each atom
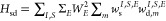
3where only contributions from the orbitals of gold atoms were considered. An example graph of the DOS for a bare gold and ligated structure decomposed into s and d states is included in Figure S1.

## Data Availability

The phosphine-stabilized gold structures are all publicly accessible on MPContribs at the following link: https://contribs.materialsproject.org/projects/auph3.

## Supporting Information Available

Distribution of structures in the data set, density of states with and without ligation, s–d hybridization calculations for planar vs nonplanar structures, binding energies to corners vs edges/faces of clusters, a detailed phase diagram for the gold size range of *n* = 3–12, detailed relative energy diagrams with renderings of low-energy structures, ligand binding energies, hidden ground states discovered as a result of this study, and experimental reference structures with their electron counts (PDF)

## Notes

Caitlin A. McCandler, Jakob C. Dahl, Kristin A. Persson; Phosphine-stabilized hidden ground states in gold clusters investigated via a Au_*n*_(PH_3_)_*m*_ database. 2022. *ChemRxiv*. 10.26434/chemrxiv-2022-q1hj2 (accessed July 21, 2022).
