# Supplementary material for: Phosphine-Stabilized Hidden Ground States in Gold Clusters Investigated via a Aun(PH3)m Database
Source: ACS Nano. 2022 Dec 30;17(2):1012–21. doi: 10.1021/acsnano.2c07223 (PMC9879275; doi:10.1021/acsnano.2c07223)
Supplement: Supplementary file 1 — nn2c07223_si_001.pdf [file nn2c07223_si_001.pdf]

Phosphine-stabilized hidden ground states in gold clusters investigated via a $Au_n(PH_3)_m$ database: Supplementary Information

Caitlin A. McCandler[1,2], Jakob C. Dahl[2,3,4], Kristin A. Persson[1,4,*]

[1]Department of Materials Science, University of California Berkeley, CA 94720, USA
[2]Materials Science Division, Lawrence Berkeley National Laboratory, Berkeley, CA 94720, USA
[3]Department of Chemistry, University of California Berkeley, CA 94720, USA
[4]Molecular Foundry, Lawrence Berkeley National Laboratory, Berkeley, CA 94720, USA
[*]Corresponding author: Kristin Persson, kapersson@lbl.gov

November 17, 2022

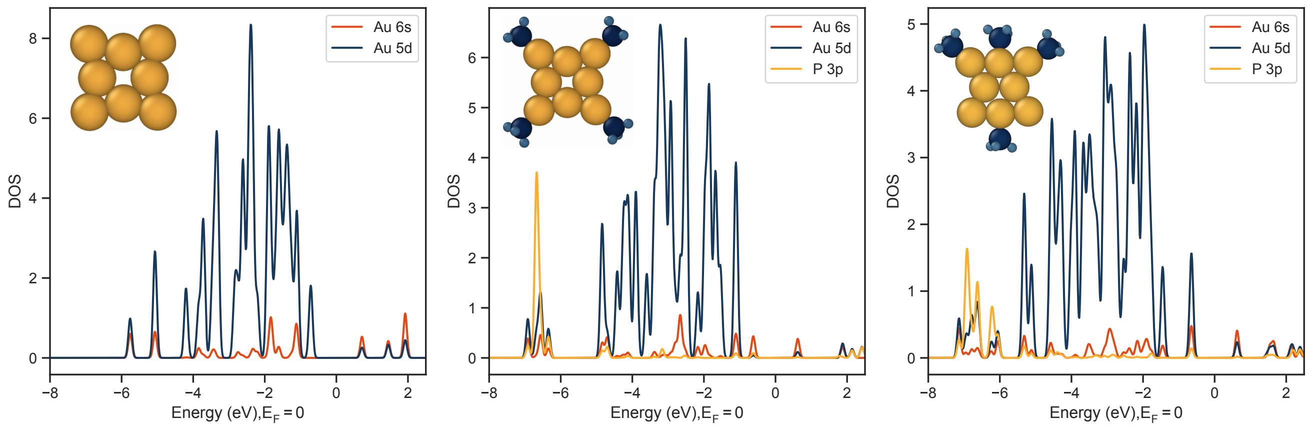

Figure S1: Density of states (DOS) of representative examples of a (left) bare gold cluster, and (middle, right) phosphine-stabilized gold clusters. The middle structure has an energy of -84.47 eV and the right structure has an energy of -83.73 eV. Only s and d orbitals from Au atoms and p orbitals from P atoms are included in the DOS. The DOS were smeared with Gaussian broadening of 0.1 eV applied around the discrete electronic energy levels for easier visualization. The DOS for the bare structure on the left matches results obtained in other studies[1,2].

References

[1] Grönbeck, H.; Broqvist, P. Comparison of the bonding in Au 8 and Cu 8 : A density functional theory study. *Phys. Rev. B* **2005**, *71*, 073408.

[2] Xiong, X.-G.; Xu, W.-H.; Li, J.; Pyykkö, P. Aspects of bonding in small gold clusters. *Int. J. Mass Spectrom.* **2013**, *354-355*, 15–18.

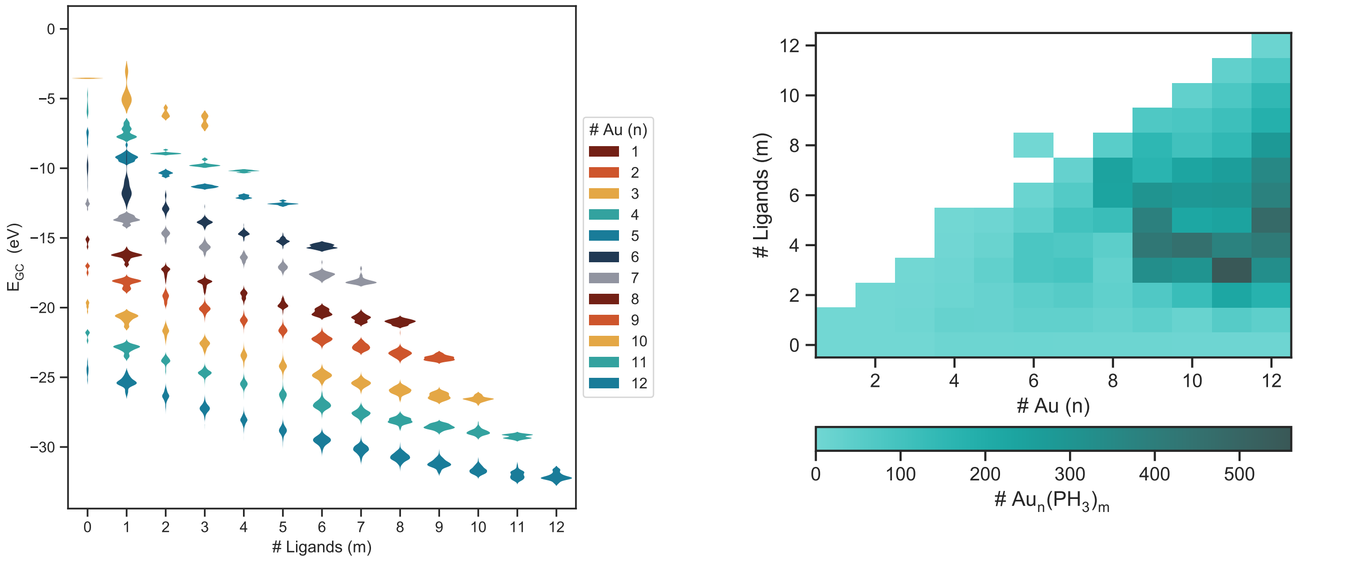

(a) Energetic distribution in the grand canonical energy (E_{GC}) formulation

(b) The number of $Au_n(PH_3)_m$ structures of each size, n and m

Figure S2: Distributions of the $Au_n(PH_3)_m$ dataset

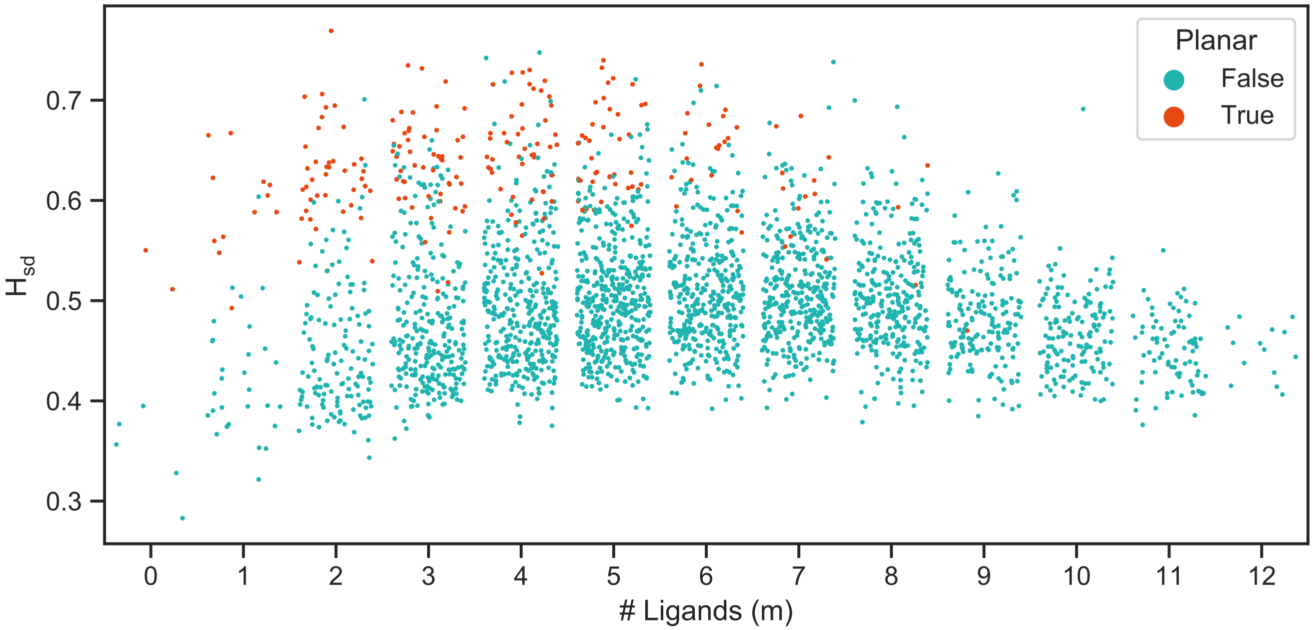

Figure S3: S-d hybridization is higher for planar structures. This is a representative example of the degree of hybridization in all planar and non-planar structures with n=12, results with other cluster sizes are similar.

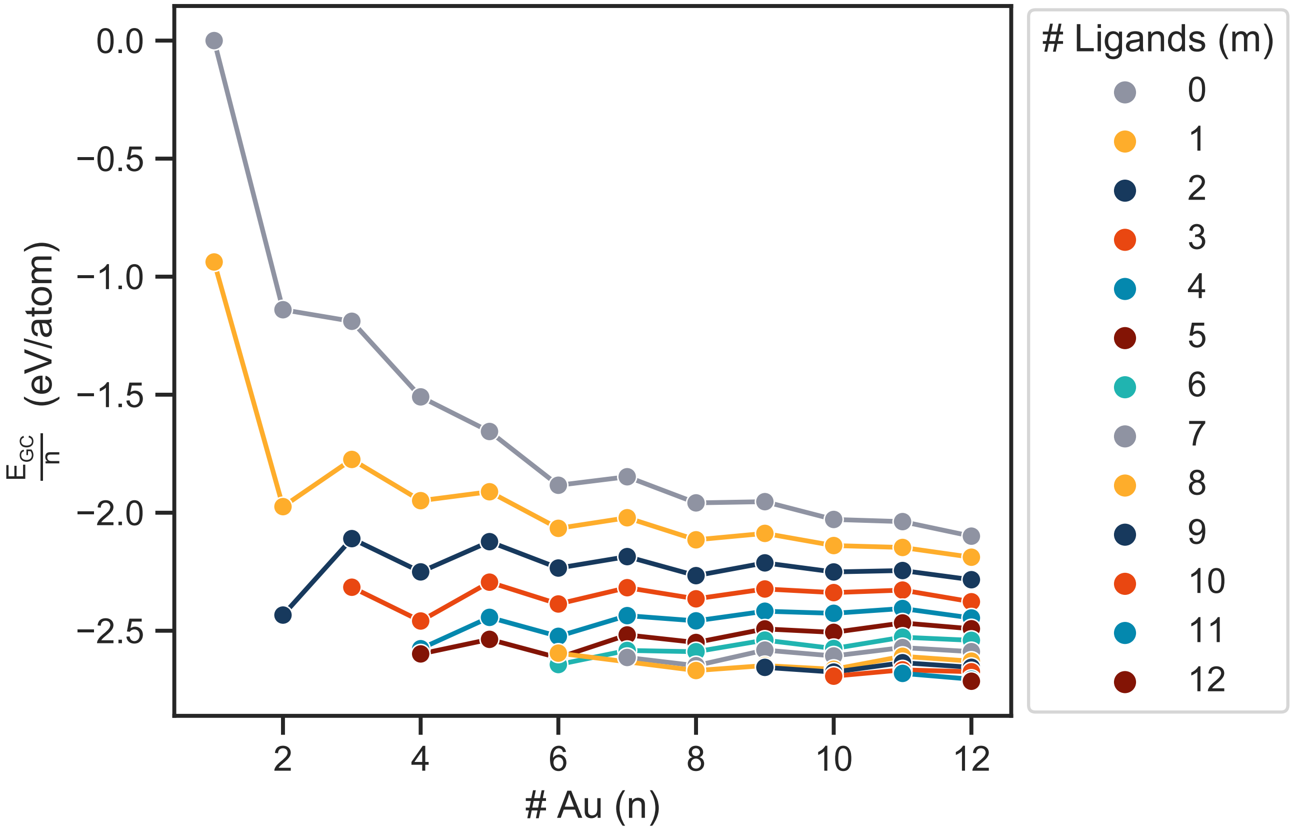

Figure S4: Grand canonical energy per atom of all the clusters, showing odd-even alternation in energies.

Au (n)	PH$_3$ Ligand Binding Energy (eV)											
	1st	2nd	3rd	4th	5th	6th	7th	8th	9th	10th	11th	12th
Au$_1$	0.967											
Au$_2$	1.715	0.961										
Au$_3$	1.819	1.070	0.681									
Au$_4$	1.836	1.322	0.905	0.536								
Au$_5$	1.367	1.147	0.968	0.810	0.604							
Au$_6$	1.159	1.156	0.981	0.909	0.674	0.398						
Au$_7$	1.313	1.276	0.974	0.973	0.692	0.625	0.470					
Au$_8$	1.319	1.283	0.851	0.810	0.888	0.776	0.542	0.405				
Au$_9$	1.286	1.212	1.087	0.991	0.739	0.799	0.782	0.609	0.388			
Au$_{10}$	1.202	1.216	1.172	1.105	0.838	0.679	0.908	0.565	0.280	0.571		
Au$_{11}$	1.152	1.192	1.005	0.929	1.019	0.897	0.746	0.676	0.429	0.661	0.496	
Au$_{12}$	1.123	1.244	1.220	0.982	0.813	1.042	0.776	0.635	0.567	0.633	0.446	0.388

Table S1: Ligand binding energies. Calculated using Eq. 2 with μ_{PH_3} = -15.62 eV

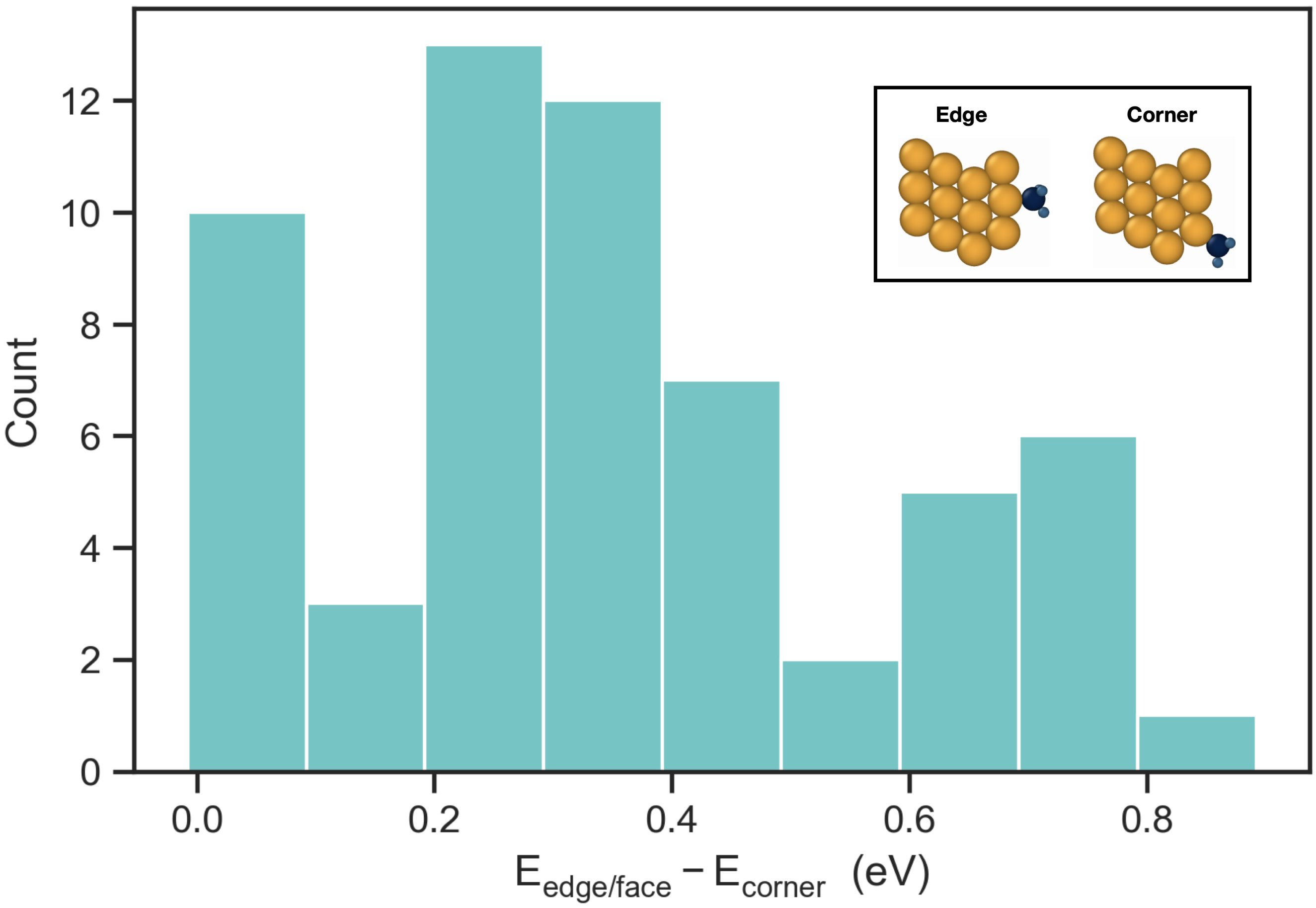

Figure S5: Binding energy preference to corners over edges and faces of gold. Corner binding sites are defined as having no gold-gold-gold angles greater than 140°.

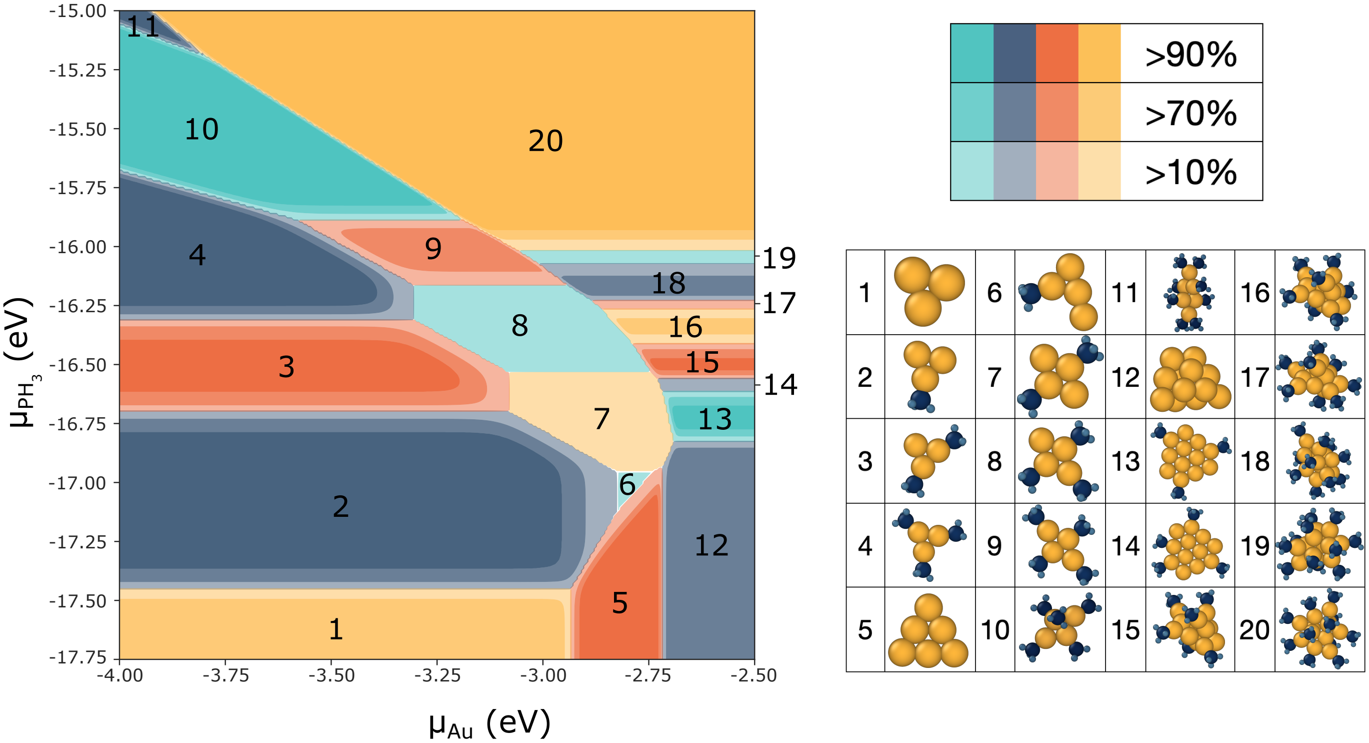

Figure S6: Phase diagram without considering monomer and dimers. The phase diagram was created by finding the most stable structure in the dataset (excluding the monomer and dimer) at that given chemical potential. The stable species are shown on the right. The fraction of the solution product that they would expect to have is calculated at 300K via a Boltzmann population.

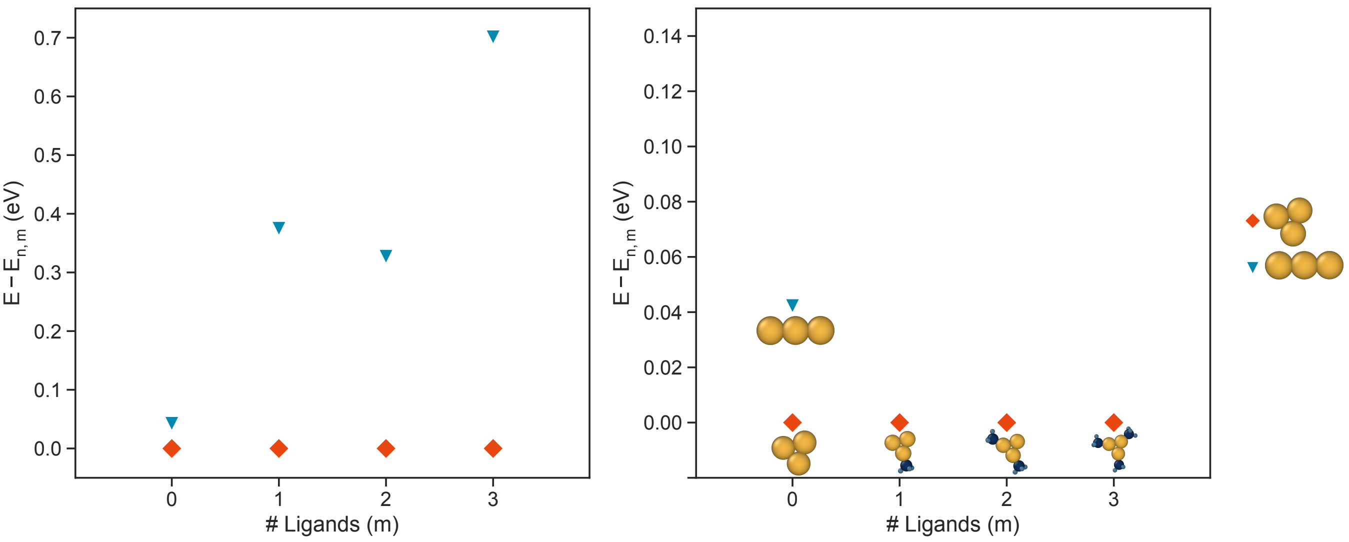
E − E_{n, m} (eV)
Ligands (m)
E − E_{n, m} (eV)
Ligands (m)

(a) n=3

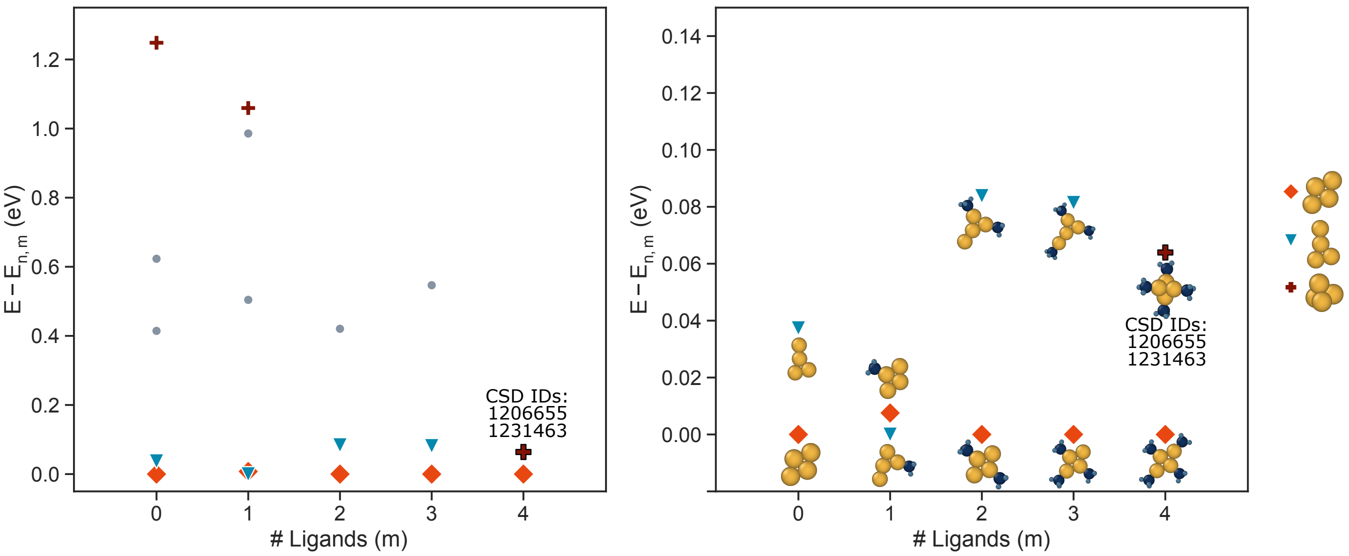
E − E_{n, m} (eV)
Ligands (m)
CSD IDs:
1206655
1231463
E − E_{n, m} (eV)
Ligands (m)
CSD IDs:
1206655
1231463

(b) n=4

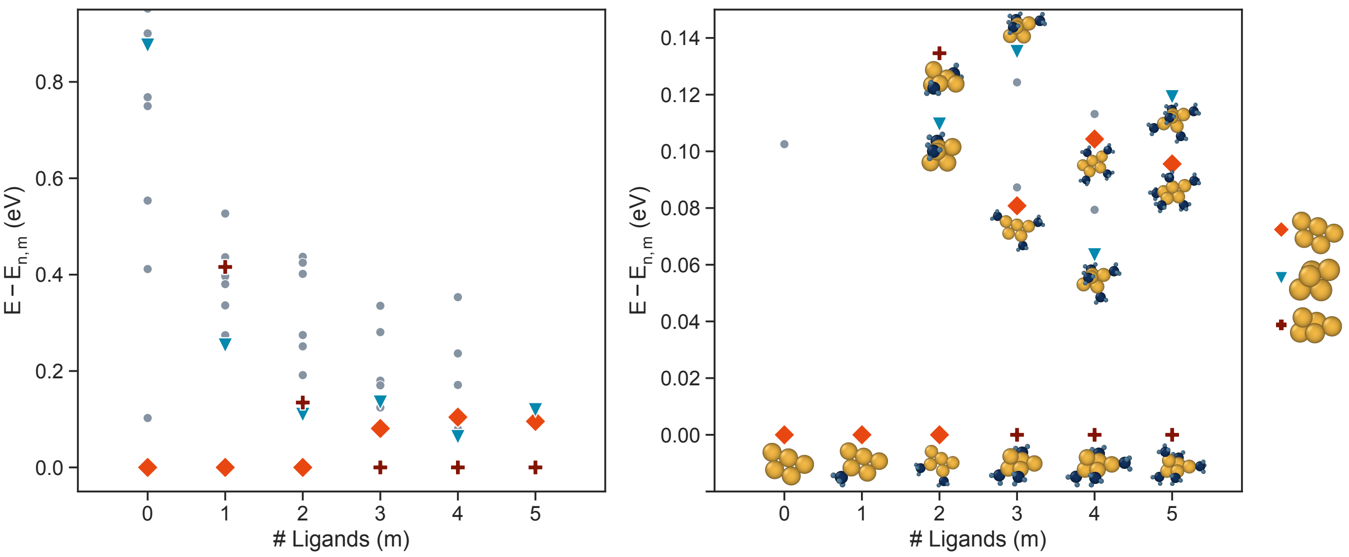
E − E_{n, m} (eV)
Ligands (m)
E − E_{n, m} (eV)
Ligands (m)

(c) n=5

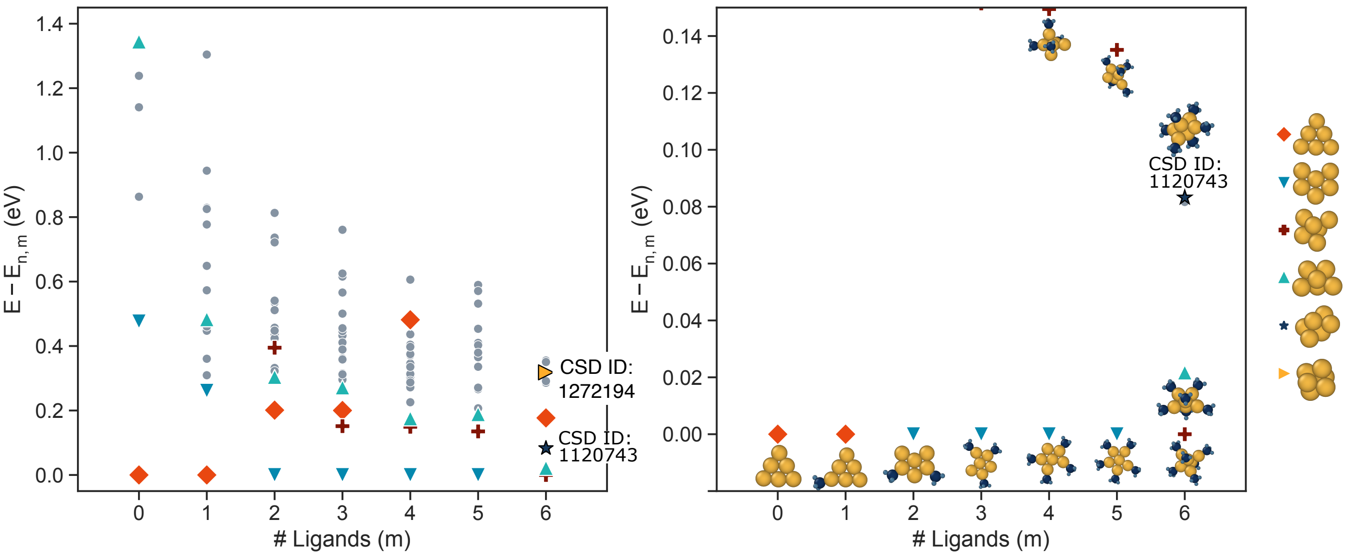
E − E_{n,m} (eV)
Ligands (m)
CSD ID:
1272194
CSD ID:
1120743
CSD ID:
1120743

(d) n=6

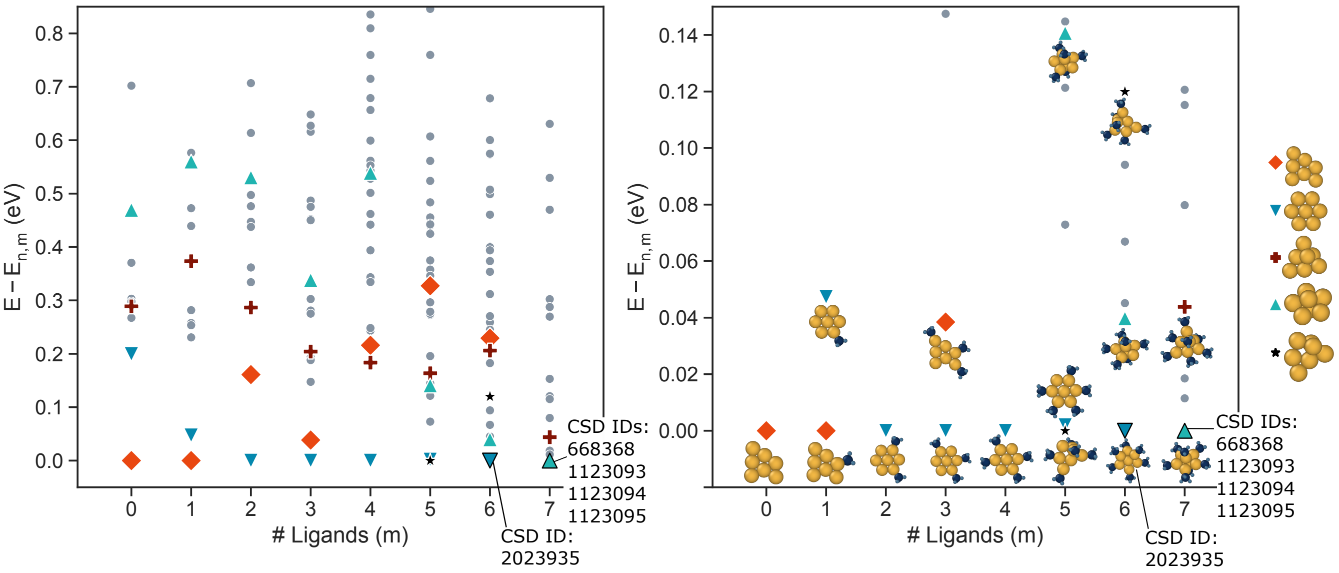
E − E_{n,m} (eV)
Ligands (m)
CSD IDs:
668368
1123093
1123094
1123095
CSD ID:
2023935
CSD IDs:
668368
1123093
1123094
1123095
CSD ID:
2023935

(e) n=7

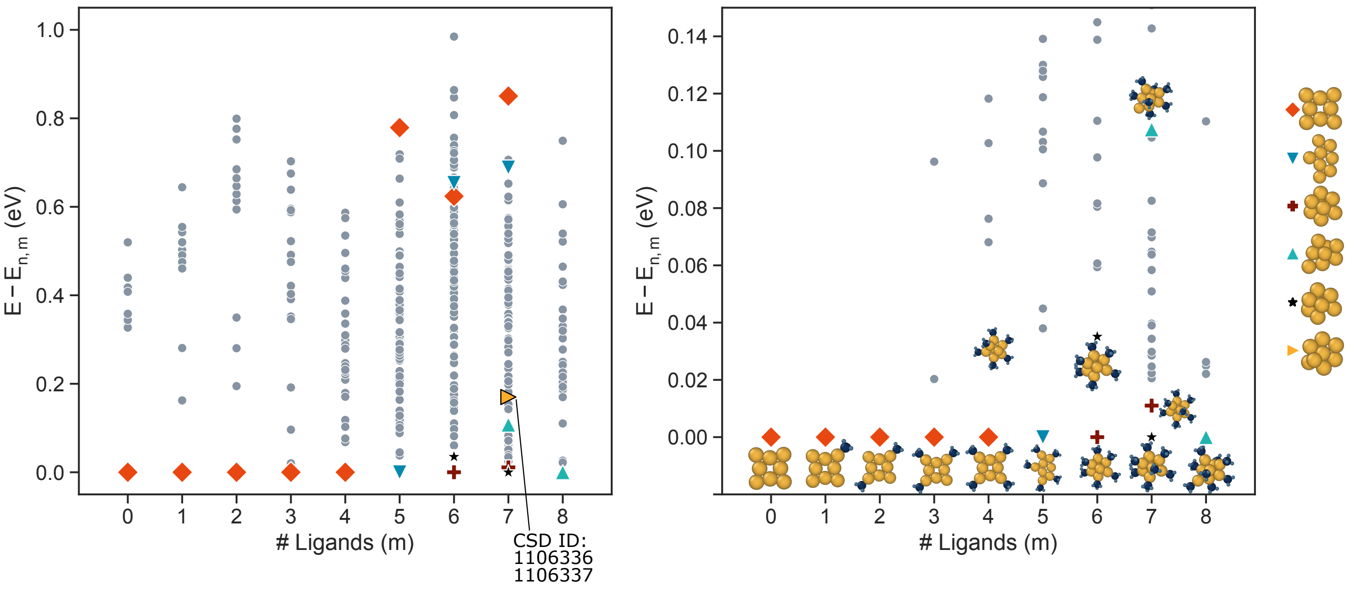
E − E_{n,m} (eV)
Ligands (m)
CSD ID:
1106336
1106337

(f) n=8

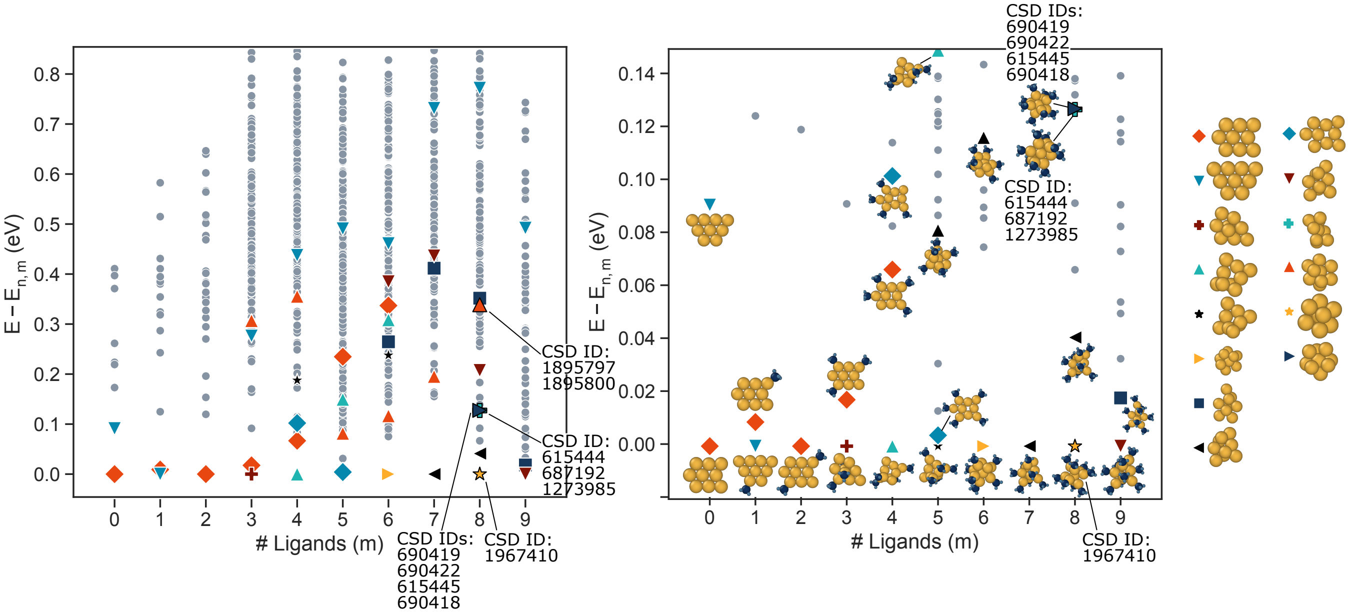

(g) n=9

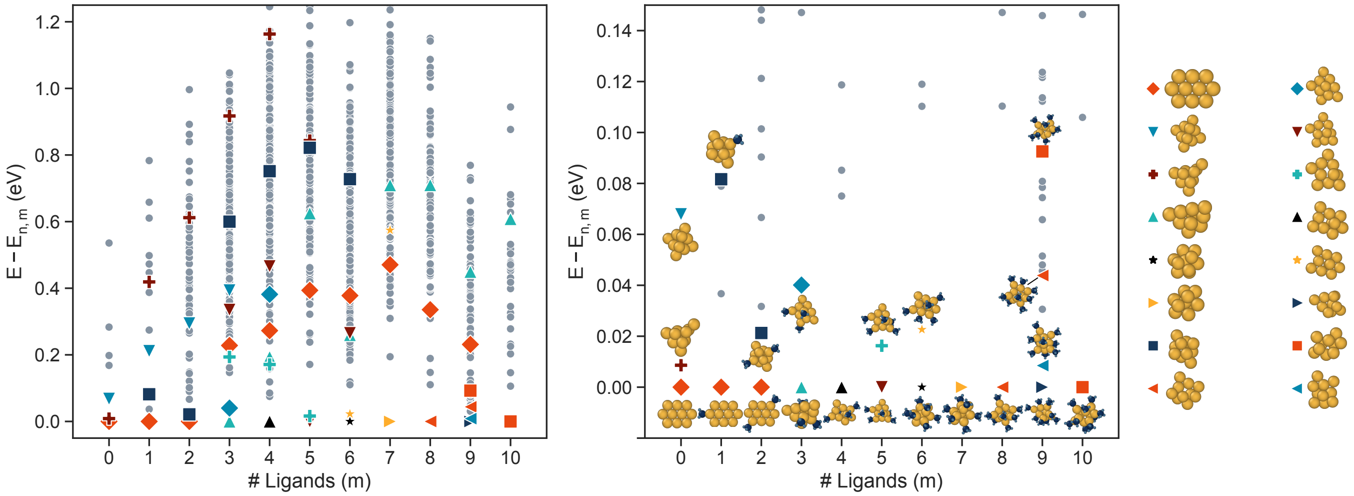

(h) n=10

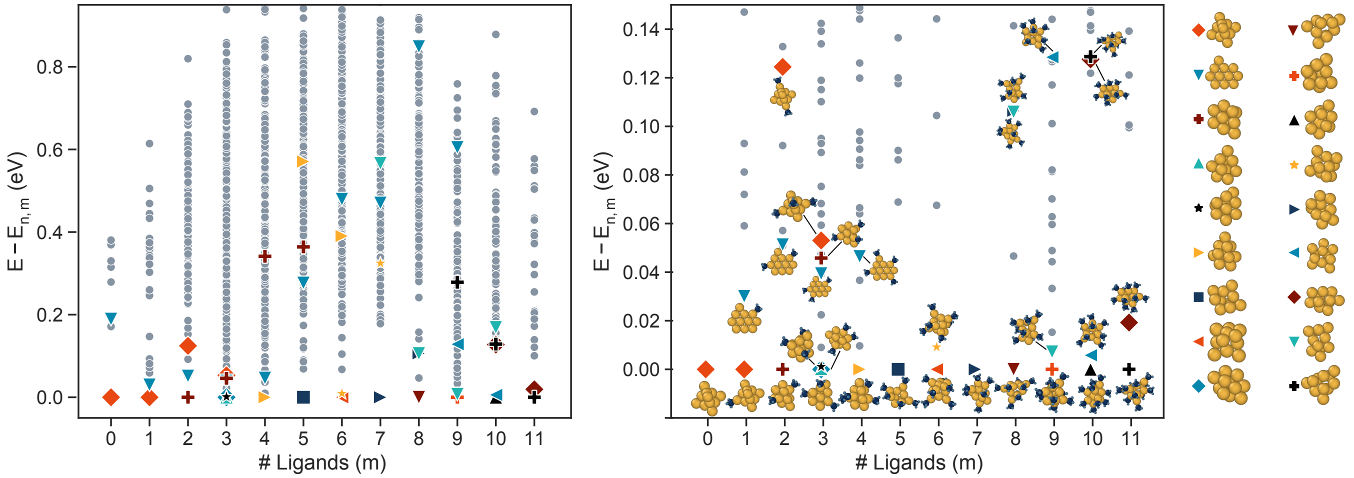

(i) n=11

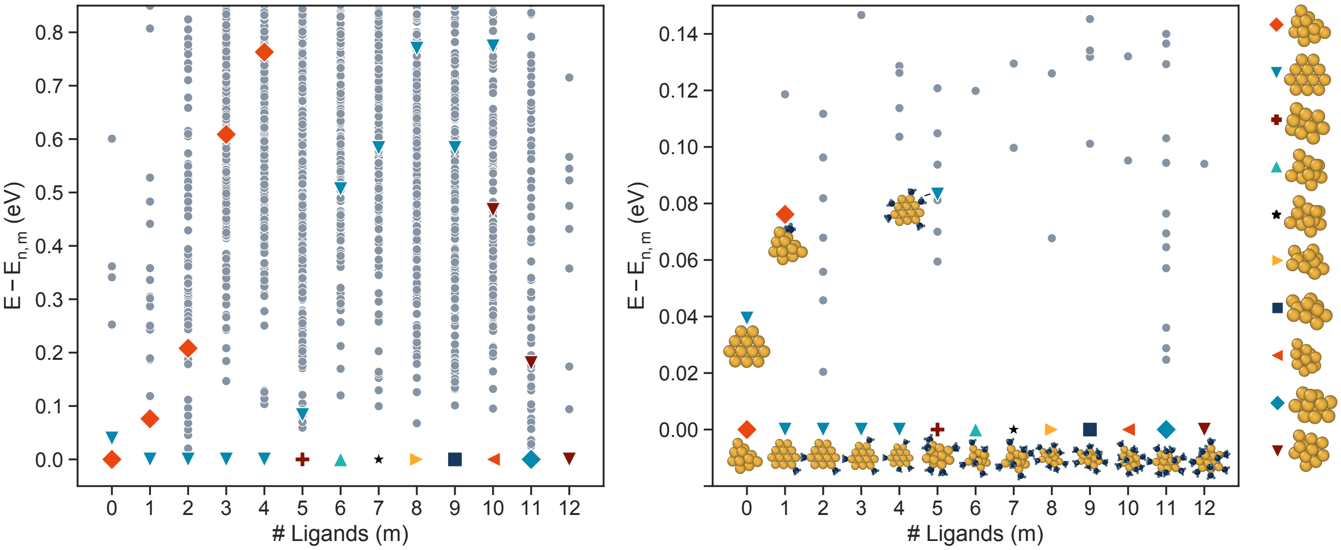

(j) n=12

Figure S7: Energies of $Au_n(PH_3)_m$ isomers for varying number of ligands, m. The energies are referenced to the minimum value for each m, $E_{n,m}$. Each point represents a unique gold kernel with the optimal ligand configuration for the given m; relevant gold kernels are highlighted in order to demonstrate how relative energies change with ligation. Experimental structures from literature sourced from the CSD are denoted with black outlines and their CSD IDs. Note that equivalent gold kernels are defined as having the same gold-gold bonding with a bond length cutoff of 3.2Å.

Au (n)	Ligands (m)	Unique ID	Image	Au (n)	Ligands (m)	Unique ID	Image
5	3	84	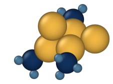	11	2	163	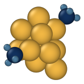
5	4	109	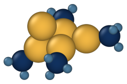	11	3	407	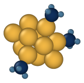
5	5	121	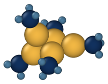	11	4	1039	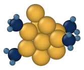
8	6	546	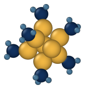	11	7	1953	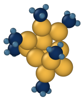
8	7	902	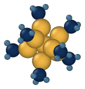	11	9	2317	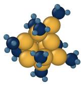
8	8	923	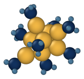	11	11	2554	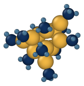
9	3	185	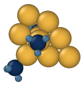	12	5	1345	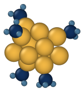
9	6	1212	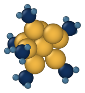	12	6	1479	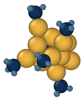
9	7	1532	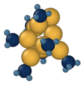	12	7	1831	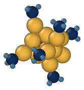
9	9	1832	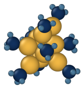	12	9	2558	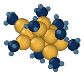
10	3	249	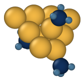	12	10	2858	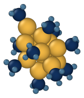
10	4	640	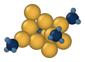	12	12	3043	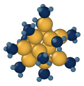
10	10	2030	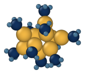				

Table S2: Hidden ground states. These structures are all ground states for different size combinations of n and m. When their ligands are removed and they are geometrically relaxed again with DFT, their bonding changes, indicating that the ligands change the potential energy surface of gold to have different local energy minima, which we call hidden ground states.

CSD ID	Au (n)	Ligands (m)	Charge (+)	Total e- count
1206655	4	4	2	10
1231463	4	4	2	10
1120743	6	6	2	16
1272194	6	6	2	16
2023935	7	6	1	18
668368	7	7	1	20
1123094	7	7	1	20
1123093	7	7	1	20
1123095	7	7	1	20
1106337	8	7	2	20
1106336	8	7	2	20
1895800	9	8	3	22
1895797	9	8	3	22
615444	9	8	3	22
687192	9	8	3	22
1273985	9	8	3	22
1967410	9	8	3	22
690419	9	8	3	22
690422	9	8	3	22
615445	9	8	3	22
690418	9	8	3	22

Table S3: The experimental structures that were used as references in this study and their total electron counts, with the assumption that each gold donates 1 electron and each phosphine donates 2 electrons to the superatom.
